# CaMKII in Regulation of Cell Death During Myocardial Reperfusion Injury

**DOI:** 10.3389/fmolb.2021.668129

**Published:** 2021-06-01

**Authors:** Yingjie Yang, Kai Jiang, Xu Liu, Mu Qin, Yaozu Xiang

**Affiliations:** ^1^ Department of Cardiology, Shanghai Chest Hospital, Shanghai Jiao Tong University, Shanghai, China; ^2^ Shanghai East Hospital, School of Life Sciences and Technology, Tongji University, Shanghai, China

**Keywords:** CaMKII, posttranslational modification, cardiomyocyte death, I/R injury, cardiovascular disease

## Abstract

Cardiovascular disease is the leading cause of death worldwide. In spite of the mature managements of myocardial infarction (MI), post-MI reperfusion (I/R) injury results in high morbidity and mortality. Cardiomyocyte Ca^2+^ overload is a major factor of I/R injury, initiating a cascade of events contributing to cardiomyocyte death and myocardial dysfunction. Ca^2+^/calmodulin-dependent protein kinase II (CaMKII) plays a critical role in cardiomyocyte death response to I/R injury, whose activation is a key feature of myocardial I/R in causing intracellular mitochondrial swelling, endoplasmic reticulum (ER) Ca^2+^ leakage, abnormal myofilament contraction, and other adverse reactions. CaMKII is a multifunctional serine/threonine protein kinase, and CaMKIIδ, the dominant subtype in heart, has been widely studied in the activation, location, and related pathways of cardiomyocytes death, which has been considered as a potential targets for pharmacological inhibition. In this review, we summarize a brief overview of CaMKII with various posttranslational modifications and its properties in myocardial I/R injury. We focus on the molecular mechanism of CaMKII involved in regulation of cell death induced by myocardial I/R including necroptosis and pyroptosis of cardiomyocyte. Finally, we highlight that targeting CaMKII modifications and cell death involved pathways may provide new insights to understand the conversion of cardiomyocyte fate in the setting of myocardial I/R injury.

## Introduction

Cardiovascular disease is the leading cause of death worldwide (Heusch, 2020), which accounts for about 30% of all deaths. Ischemic heart disease accounts for nearly half of all cardiovascular deaths in low- and middle-income countries (Zhou et al., 2016; Prabhakaran et al., 2018; Zhao et al., 2019). During myocardial infarction (MI), the ischemia and hypoxia status due to coronary artery obstruction results in the injury and death of cardiomyocyte. Therefore, timely thrombolytic therapy or percutaneous coronary intervention to restore coronary blood flow is the effective method to reduce acute MI injury and limit MI area (Hausenloy and Yellon, 2008; Hausenloy and Yellon, 2013; Heusch, 2020). However, the reperfusion of MI leads to cardiac injury, such as abnormal cardiac electrical activity, myocardial stunning, microvascular obstruction, and lethal myocardial reperfusion injury (Hausenloy and Yellon, 2013). From the point of view of cell biology, the response of cardiomyocyte during myocardial I/R includes activated immune response, organelle dysfunctions, and shifted metabolic pathways (Hausenloy and Yellon, 2013). The key procedure in reperfusion after MI is the overproduction of ROS by abnormal mitochondrial dynamic in endothelial cell and cardiomyocyte (Wang et al., 2020b) and Ca^2+^ overload (Talukder et al., 2009). The mitochondrial homeostasis can be maintained by mitochondrial quality control (MQC) by mitochondrial fission, fusion, or mitophagy (Wang and Zhou, 2020), while during cardiac microvascular I/R, MQC defection leads to ROS overproduction (Wang et al., 2020a), which recruits neutrophils to the lesion (Hausenloy and Yellon, 2013) and triggers the NF-κB inflammatory pathway (Morgan and Liu, 2011). ROS can also lead to Ca^2+^ overload by ER stress, which is the main cause of mitochondrial permeability transition pore (mPTP) opening and myofilament hyper-contraction (Hausenloy and Yellon, 2013). The opening of mPTP results in the increased ROS production, which forms a positive feedback between ROS and Ca^2+^ overload until cell death. CaMKII, as a substrate of Ca^2+^, is greatly involved in the ROS and Ca^2+^ overload feedback. Therefore, it is of great significance to decipher the mechanism of CaMKII involved in regulation of cell death during myocardial I/R injury, contributing to potential drug targets discovery.

**FIGURE 1:**
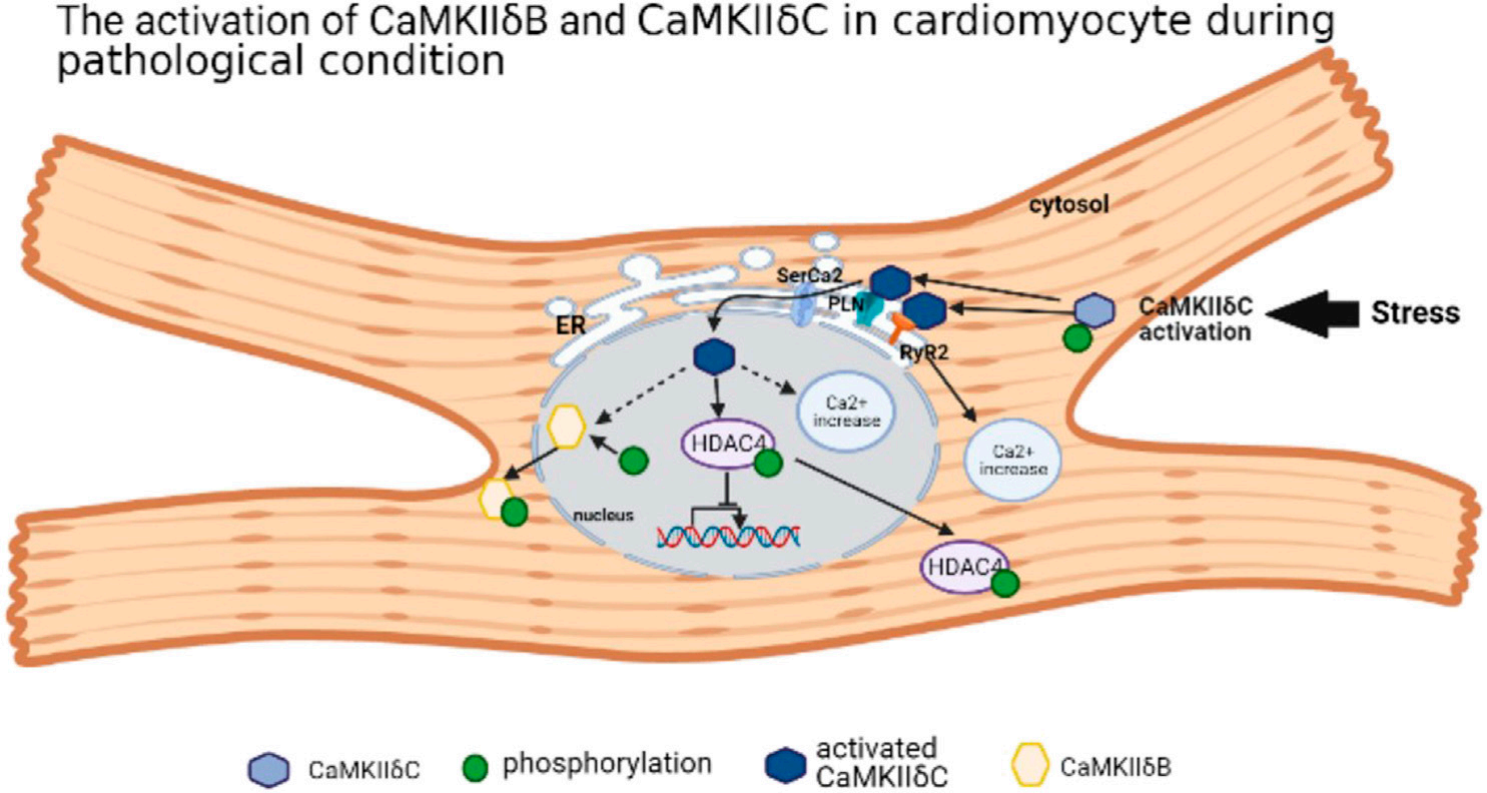
The activation of CaMKIIδB and CaMKIIδC in cardiomyocyte during pathological condition.

Ca^2+^/calmodulin (CaM)-dependent protein kinase II (CaMKII) is a multifunctional serine/threonine kinase with four subtypes, including CaMKIIα, CaMKIIβ, CaMKIIγ, and CaMKIIδ (Erickson et al., 2011; Gray and Heller Brown, 2014). CaMKIIα and CaMKIIβ are mainly expressed in the nervous system, closely related to memory development (Mayford et al., 1996), while CaMKIIγ and CaMKIIδ are widely distributed in various organs and tissues (Gray and Heller Brown, 2014). In cardiomyocytes, CaMKIIδ is a dominant subtype (Hund et al., 2010; Erickson et al., 2011; Gray and Heller Brown, 2014). The CaMKII monomer consists of three domains: the N-terminal catalytic domain, the C-terminal binding domain, and the intermediate regulatory domain (Erickson et al., 2011; Gray and Heller Brown, 2014). The variant region (variant domain) locates in the intermediate regulatory domain, whose composition differs according to different CaMKII splicing variants. Taking CaMKIIδ as an example, there are 11 CaMKIIδ variants, among them four variants are located in the heart, including CaMKIIδA, CaMKIIδB, CaMKIIδ C, and CaMKIIδ913. Most studies focus on CaMKIIδB and CaMKIIδC because of its opposite roles in cardiomyocyte. With the nuclear localization sequence (NLS), CaMKIIδB splicing variant mainly locates in the nucleus, while phosphorylation at specific locus in the NLS may prevent the nuclear transport of CaMKIIδB (Srinivasan et al., 1994; Heist et al., 1998; Gray and Heller Brown, 2014). CaMKIIδC is in the cytoplasm due to the absence of NLS (Srinivasan et al., 1994). CaMKIIδA locates in the T-tubule, sarcolemmal, and nuclear membrane. As an embryonal CaMKII type, CaMKIIδA may strengthen the L-type calcium current for contraction in newborn cardiomyocytes (Beckendorf et al., 2018). It is almost absent in adult cardiomyocytes, but the upregulation of CaMKIIδA triggered by MI-induced HF or chronic HF leads to the hypertrophy or death of cardiomyocyte (Gui et al., 2018). For CaMKIIδ9, its activation also leads to cardiomyocyte injury by inhibiting DNA repair (Zhang M. et al., 2019). Under certain cardiac pathology condition, for example, pression overload or I/R stress, CaMKIIδC is activated with the suppression of CaMKIIδB (Gray et al., 2017; Ljubojevic-Holzer et al., 2020). However, CaMKIIδB is highly expressed in the hypertrophied cardiomyocyte model induced by transverse aortic constriction (TAC), which is degraded by calpain-2 to trigger the mitochondrial apoptosis pathway (Sheng et al., 2015). CaMKIIδB is located in the nucleus, but it can also be expressed in the endoplasmic reticulum (ER), membrane, cytosol, and mitochondria (Mishra et al., 2011). Under stress, CaMKIIδC is activated by autophosphorylation, and it migrates to the ER and activates two ER receptors: ryanodine receptor 2 (RyR2) and phospholamban (PLN), which were phosphorylated by activated CaMKIIδC (Beckendorf et al., 2018) resulting in the Ca^2+^ leak into cytoplasm. Furthermore, activated CaMKIIδC governs sarcoplasmic reticulum Ca^2+^-ATPase2 (SerCa2) and stimulates PLN on the nuclear membrane, which enables autophosphorylated CaMKIIδC to transfer into the nucleus. Activated CaMKIIδC binds and phosphorylates histone deacetylase 4 (HDAC4) protein, causing the disability of nuclear transcription and nuclear location. In addition, CaMKIIδC activation also leads to increased intranuclear Ca^2+^ and aggravates nuclear disorder (Ljubojevic-Holzer et al., 2020). CaMKIIδB is also autophosphorylated at the locus Ser332 in its NLS, contributing to its migration to the cytosol (Gray and Heller Brown, 2014). Thus, different subtypes of CaMKIIδ have their own effect on physiological and pathological roles in cardiomyocyte (Figure 1).

A series of studies have shown that myocardial I/R is significantly alleviated by inhibiting the activity of CaMKII either by drug inhibition, including KN93 (Mattiazzi et al., 2007), AIP (Vila-Petroff et al., 2007; Salas et al., 2010), or by gene inhibition (Ling et al., 2013). CaMKII, as a protein kinase, has a series of target proteins in cardiomyocytes. Through posttranslational modification (majority phosphorylation) of these target proteins, CaMKII is involved in the regulation of cardiomyocyte ion homeostasis, contraction, inflammatory response, and programmed cardiomyocyte death. In CaMKIIδ knockout mice in cardiomyocyte, the inflammatory response was suppressed (Willeford et al., 2018), and the apoptosis and cardiac hypertrophy were reduced significantly (Daniels et al., 2015), revealing a protection effect of inhibiting CaMKIIδ in cardiomyocyte.

**FIGURE 2:**
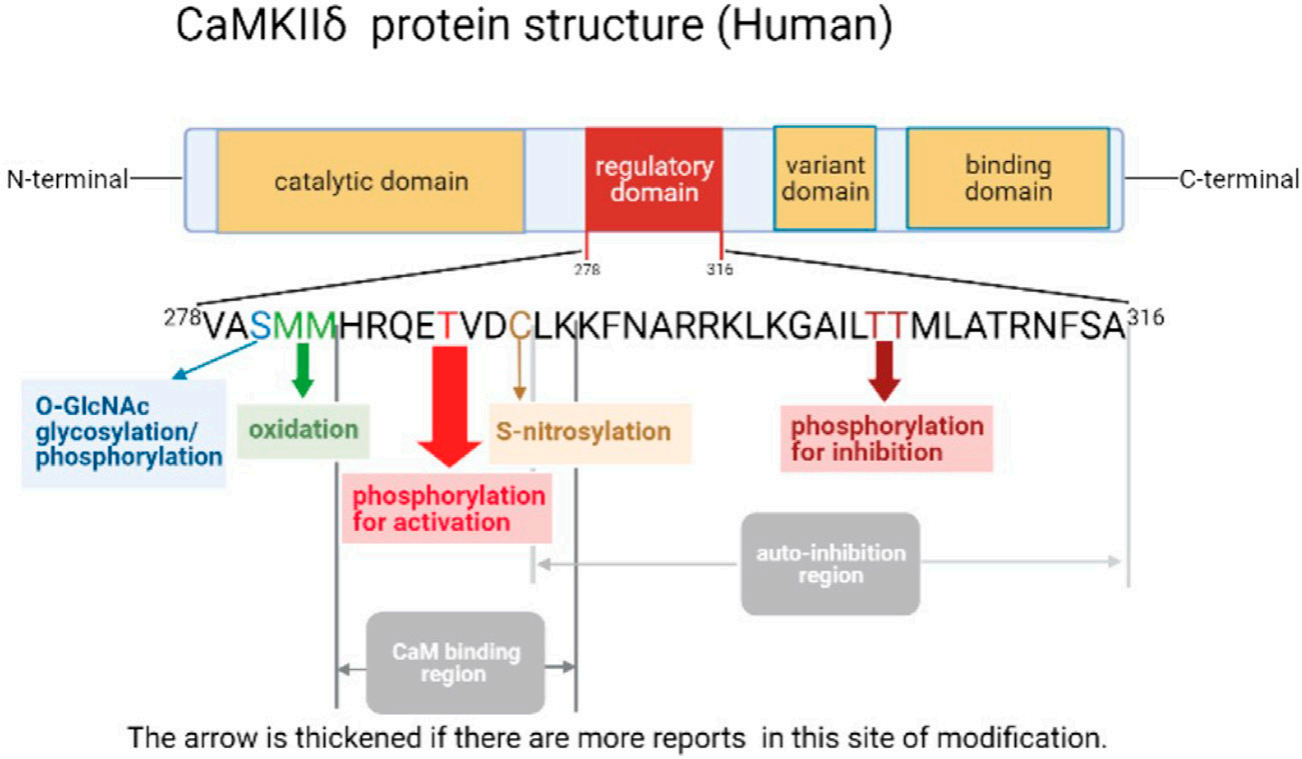
The effect of CaMKIIδ related pathway in cardiomyocyte during I/R.

In brief, the majority way to activate CaMKII in cardiomyocyte is by stimulating β1-adrenergic receptor (β1-AR) (Pereira et al., 2013) and increasing Ca^2+^ concentration [often by L-type Ca^2+^ channel (LTCC)] (Beckendorf et al., 2018), but intriguingly, the blockage of β1-AR cannot inactive CaMKII (Dewenter et al., 2017). Moreover, its activation is closely related to ER stress in I/R. By phosphorylating RyR2 Ser2814 and PLN Ser16 and Thr17 loci, the SerCA2 was opened, leading to ER stress and Ca^2+^ leakage (Netticadan et al., 2000). A large amount of Ca^2+^ entering the cytoplasm directly induced contracture of myofilaments and diastolic dysfunction (Boontje et al., 2011). Furthermore, a large amount of Ca^2+^ in cytoplasm is transported into mitochondria by mitochondrial Ca^2+^ unidirectional transporter (MCU), leading to the opening of (mPTP) to cause cardiomyocyte death (Joiner et al., 2012). In addition, mitochondrial stress produces more intracellular ROS and this stimulates CaMKII and forms a positive feedback, resulting in the accumulation of a large number of intracellular Ca^2+^ until cell death (Luo et al., 2019). Therefore, from the perspective of organelles, the activation of CaMKII in I/R results in the stress of both ER and mitochondria, the increase of intracellular Ca^2+^ concentration, and myofilament contracture (Figure 2). Meanwhile, the relative transmembrane ion channels are changed to affect the intracellular ion homeostasis. The detail was not mentioned in this article.

## Calmodulin-Dependent Protein Kinase II Modification During Myocardial I/R Injury

**FIGURE 3:**
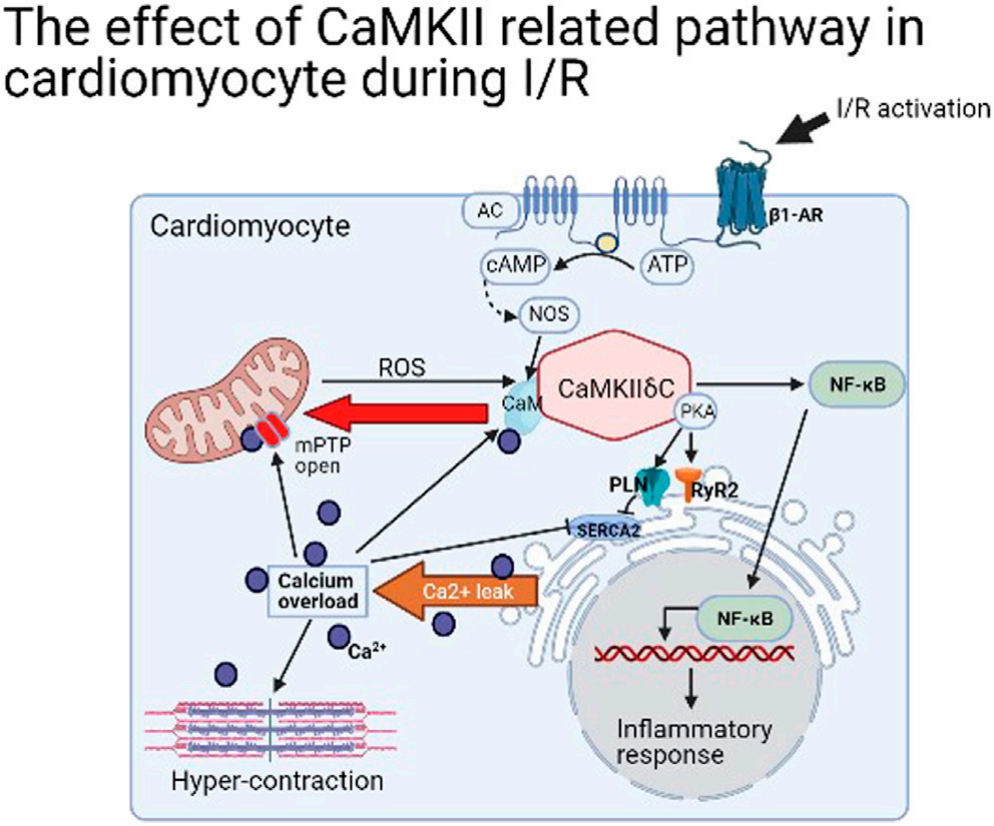
Schema of human CaMKIIδ protein structure.

CaMKII’s activity is regulated by numerous proteins, with two key steps to activate CaMKII, including the conformational change by Ca^2 +^/CaM-dependent and posttranslational modification. CaMKII is a dodecamer protein assembly in the intracellular Ca^2+^ homeostasis, which possesses an auto-inhibitory structure. With the elevation of intracellular Ca^2+^ and tightly combined to the CaM for activation, thus by binding to the regulatory domain, the conformation of dodecamer changes with the N-terminal catalytic domain closing to ATP and substrate protein. Full exposure to the intermediate regulatory domain endows CaMKII prone to oxidation and autophosphorylation (Erickson et al., 2011; Rokita and Anderson, 2012). The catalytic domain transfers ATP’s phosphoric group to CaMKII Ser287 locus (subtype α is Ser286) to change it as an active state, and then increases the binding force of phosphorylated-CaMKII to Ca^2+^/CaM, which is known as Ca^2+^/CaM-dependent direct activation, the most common posttranslational modification of CaMKII (Rokita and Anderson, 2012). Self-phosphorylated CaMKII can be dephosphorylated by protein phosphatases (including PP1 or PP2A) to restore the self-inhibited state, which are potential targets in cardiac disease like HF, arrhythmia, and MI (Strack et al., 1997; Fischer et al., 2018; El Refaey et al., 2019). While autophosphorylation of Thr305 locus in CaMKIIγ (Munevar et al., 2008) (Thr305 and Thr306 in CaMKIIα) inhibits the binding ability between CaMKII and Ca^2+^/CaM, resulting in decreased CaMKII activity (Cook et al., 2021). Moreover, CaMKII can also be activated by the direct oxidation of CaMKII Met281 and Met282 (α-subtype is CM 280/281) by increased reactive oxygen species (ROS) in cardiomyocytes. This activation manner still requires the initial binding of the Ca^2+^/CaM complex to the CaMKII regulatory domain to release the self-inhibitory structure, and the oxidized component is ROS produced by pathological stimuli or factors such as hyperglycemia, activation of the renin–angiotensin–aldosterone system (RAAS), MI, or heart failure (HF) (Erickson et al., 2008; Luo et al., 2013). As methionine oxidized reductase, methionine sulfone reductase A (MsrA) reverses the oxidative modification of CaMKII, which is a potential drug target to reduce the production of ox-CaMKII (Erickson et al., 2008). Both phosphorylation and oxidation modifications of CaMKII are present in cardiomyocytes under different physiological or pathological conditions. For example, during MI, the expression of CaMKII is significantly increased; meanwhile, aldosterone upregulates its expression through oxidation and phosphorylation, which leads to the deterioration of MI injury (He et al., 2011). Furthermore, by reducing necroptosis key protein RIP3, the activation of CaMKII, both of oxidation and phosphorylation, is suppressed in I/R or doxorubicin treatment cardiac injury (Zhang et al., 2016). In 2013, oxygen-linked acetylglucosamine transferase (OGT) glycosylation CaMKII Ser280 (α-subtype Ser279) and the formation of β-N-acetylglucosamine modification at O-site were demonstrated. This modification of CaMKII occurs in response to high-glucose stimulation (Erickson et al., 2013), while oxygen-linked acetylglucosaminase (OGA) sponges the glycosylation at the Ser280 of CaMKII, thus reversing the glycosylation modification of CaMKII (Zou et al., 2012). In addition, CaMKII can also be activated by nitrosylation in cardiomyocyte. Among them, β-adrenergic receptors (β-AR) induce the nitric oxide (NO) intracellular production, which activates CaMKII by nitrosylation rather than nitrosylating other targets like RyR2, leading to ER Ca^2+^ leak and the occurrence of arrhythmias (Gutierrez et al., 2013). Through binding to Ca^2+^/CaM, NO simultaneously nitrosylates the Cys289 residue in CaMKIIα, which reduces autophosphorylation of Thr286 (Coultrap and Bayer, 2014). In the cardiomyocyte, the S-nitrosylation of Cys290 activates CaMKIIδ, contributing to downstream Ca^2+^ leak. However, it can be reversed by S-nitrosylation of Cys273 (Erickson et al., 2015). Thus, various CaMKIIδ modifications are essential in cardiac I/R injury (See Figure 3; Table 1).

**TABLE 1:** Overview of CaMKII posttranslational modification (CaMKIIδ as an example).

CaMKII posttranslational modification	Modification locus	Substrate/donor	Function
Phosphorylation	Thr287	Substrate: protein calmodulin and donor: ATP autophosphorylation	Activation
Phosphorylation	Thr306 and Thr307 maybe (no report in CaMKIIδ)	ATP autophosphorylation	Inhibition
Oxidation	Met281 and Met282	ROS	Activation
S-nitrosylation	Cys290	NO	Activation
S-nitrosylation	Cys273	NO	Inhibition
O-GlcNAc glycosylation	Ser280	OGT	Activation

### CaMKIIδ Thr287 Phosphorylation

Phosphorylation of CaMKIIδ Thr287 occurs in many physiological conditions, and almost all cardiac pathological conditions require the activation of CaMKIIδ by phosphorylation. During MI, the upregulation of CaMKII phosphorylation affected the expression and the phosphorylation of ER receptors including RyR2, PLN, and SerCa2, which leads to ER dysfunction in post-MI HF (Netticadan et al., 2000). During reperfusion, CaMKII is autophosphorylated and redistributes into the cytoplasm (Uemura et al., 2002). Through the computer mathematical model and biochemical imaging technology applying to the MI model, it was confirmed that the autophosphorylation of CaMKII in the border zone of infarction was increased, which resulted in abnormal intracellular Ca^2+^ homeostatic and influences on sodium channels to reduce upstroke velocity of action potential (Hund et al., 2008). In an abnormal cardiac contractility after MI, increased intracellular Ca^2+^ leads to the enhanced myofilament contraction ability (Zhang Y. et al., 2019). CaMKIIδC’s phosphorylation plays an important role in the filament reactivity by modulating the phosphorylation of myofilament associated protein, myosin-binding protein C (Boontje et al., 2011; Reil et al., 2020). In addition, CaMKIIδC phosphorylation can activate NF-κB–related pathways to enhance TNF-α expression in response to I/R injury (Gray et al., 2017). During MI, CaMKII phosphorylation leads to the phosphorylation of Nav1.5, a cardiac sodium channel (Howard et al., 2018), and the reverse of Ito (a cardiac potassium channel) by decreasing Kv4.3 gene (Tao et al., 2020; Tinaquero et al., 2020), inducing arrhythmias. In the case of I/R injury, CaMKII phosphorylation can also be regulated by zinc transporters or (Wang et al., 2020c) brain-derived neurotrophic factor (BDNF) (Lee et al., 2018). Thus, CaMKII phosphorylation plays a role in ER stress, intracellular ion stabilization, and contractility in cardiomyocytes.

### CaMKIIδ Met281/282 Oxidative Modification

Since 2008, ROS was found to directly oxidize CaMKII in Met281 and Met282 sites and to induce its activation (Erickson et al., 2008). Ox-CaMKII was identified to be associated with various diseases including cardiovascular disease, arrhythmia, cancer, and asthma (Anderson, 2015). Ox-CaMKII function firstly observed in cardiac disease is the induction the apoptosis of sinoatrial node (SAN) cells, contributing to sinus node dysfunction (SND). By inhibiting the NADPH oxidase in the angiotensin II (Ang II) infusion mice model, ox-CaMKII is suppressed, thus improving the survival of SAN cells, suggesting that ox-CaMKII may be the cause of SND by triggering SAN cell death (Swaminathan et al., 2011). In terms of MI, activation of the TLR/MyD88/NF-κB pathway after MI induces CaMKII oxidation, leading to myocardial cell death. Knocking out MyD88, ox-CaMKII production was inhibited to protect the adverse hypertrophy and inflammation by LPS and MI (Singh et al., 2012). In addition, increased ox-CaMKII in mitochondria contributes to sudden death in diabetic MI, while mitochondrial antioxidants inhibited ox-CaMKII and increased the number of cardiomyocyte survival (Luo et al., 2013). Ox-CaMKII also exerts an important role in the development and maintenance of atrial fibrillation (Yoo et al., 2018), (Yang et al., 2020). Recent studies showed that ROS and O-GlcNAcylation protein (OGN) are elevated in diabetic heart disease, both of which induce atrial fibrillation. However, only ROS-induced CaMKII oxidation, while OGN is dependent on CaMKII-induced atrial fibrillation not by CaMKII glycosylation (Mesubi et al., 2021). Therefore, the interaction between the oxidation and other posttranslational modifications of CaMKII in myocardial I/R injury remains to be elucidated. In fact, several inhibitors, such as protein phosphatase 1 inhibitor 1 (I1PP1) and Chinese patent medicines, also alleviate diabetes and I/R-related myocardial injury by inhibiting ox-CaMKII (Luo et al., 2019; Liu et al., 2020).

### CaMKIIδ Cys290 Nitrosylation Modification

Before the discovery of the nitrosylation modification of CaMKII, studies have demonstrated that NO and CaMKII exhibited interaction, but the site and the role have not been clarified for a long time. In ventricular arrhythmias, phosphorylation of RyR2 receptor occurs under the condition of intracellular nitroso-redox imbalance; further study showed that its phosphorylation is related to CaMKII activation (Cutler et al., 2012). An upstream event could be represented by the stimulation of β-receptor stimulation, which activates CaMKII to induce ER Ca^2+^ leakage, but the specific mechanism and pathway remain to be elucidated (Curran et al., 2014). Until 2014, the CaMKIIα S-nitrosylation was first identified (Coultrap and Bayer, 2014), and further studies illustrated that the Cys290 nitrosylation of CaMKII by NO autonomously activates CaMKII (Erickson et al., 2015). Its activation also induces phosphorylation and nitrosylation of downstream RyR2 receptors, which can be abolished by Cys273 mutation, suggesting the potential pathogenicity and inhibitory site of CaMKII protein–causing cardiomyocyte death (Erickson et al., 2015).

### CaMKIIδ Ser280 Glycosylation Modification

O-N-acetylglucosamine (O-GlcNAc) glycosylation of CaMKII mediated by O-GlcNAc transferase (OGT) is a unique form of activation that does not affect Ca^2+^/CaM-induced direct activation of CaMKII. Similar to other phosphorylation, O-GlcNAc glycosylation of CaMKII is an inducible, reversible, and dynamic posttranslational modification. In addition to OGT, another glycosidase O-GlcNAcase (OGA) also regulates the activity of CaMKII. Unlike phosphorylation mediated by huge amounts of kinases and phosphatases, the reversible modification of O-GlcNAc glycosylation is only catalyzed by glycosylation (OGT) and deglycosylation (OGA) (Erickson et al., 2013).

O-GlcNAc is closely related to the level of glucose, so CaMKII glycosylation is often modulated by the level of glycemia in physiology or pathology status. Glucose deprivation has been reported significantly to increase O-GlcNAc levels, and it is associated with decreased OGA but not with increased OGT (Zou et al., 2012). In neonatal cardiomyocytes, glucose deprivation and heat shock both increase O-GlcNAc levels, which is overturned by CaMKII inhibitor KN93, suggesting that intracellular CaMKII activation induced by Ca^2+^ plays a key role in regulating the increase in O-GlcNAc levels (Zou et al., 2012). CaMKII is highly expressed and highly active in the hearts of patients with diabetes and HF, especially CaMKII O-GlcNAc glycosylation. The first research about OGT published in 2010 showed that OGT deletion worsens the cardiac function in post-MI HF (Watson et al., 2010). In terms of hyperglycemia, the sudden elevation of glycemia results in the induction of arrhythmias due to CaMKII activation through glycosylation (Erickson et al., 2013). Further studies showed a transient increase in glycemia due to stress may increase the speed of Ca^2+^ waves and downregulate the cardiac potassium channel amplitude through CaMKIIδ-Ser280-GlcNAcylation. Moreover, chronic hyperglycemia and CaMKII activation during diabetes downregulate K^+^ channel expression and function, both of which increase sensitivity to arrhythmias possibly by O-GlcNAc glycosylation (Hegyi et al., 2020; Miura et al., 2020). However, when acute hyperglycemia was used to induce the glycosylation of CaMKII in animal experiments, only a low arrhythmic substrate was observed and atrial fibrillation was not induced (Manninger et al., 2020). Moreover, the most recent study showed that the locus of Ser280 in CaMKIIδ acts as phosphorylation rather than glycosylation in the model of AF (Mesubi et al., 2021), which should be further verified and explored in its change of conformation and function. In terms of MI or myocardial I/R, the glycosylation of CaMKII was increased in the type 2 diabetes mellitus (Wang et al., 2018). CaMKII glycosylation induces the occurrence of myocardial cell damage and a series of *in vivo* and *in vitro* experiments proved, suggesting O-GlcNAc influences myocardial I/R injury through various ways (Ngoh et al., 2010; Dassanayaka and Jones, 2014). Its various functions in the nervous system are also new fields not only in heart diseases but also in neuronal functions (Lagerlöf et al., 2016).

## Calmodulin-Dependent Protein Kinase II Involved in Inflammatory Response and Various Cardiomyocyte Cell Death Modes

### Calmodulin-Dependent Protein Kinase II and Apoptosis

As early as 1996, it was found that apoptosis was the main death mode of cardiomyocyte after MI, which accounts for more than 90% of death myocyte 2 h after MI, and the necrosis of myocardial cells reached the peak after apoptosis (Kajstura et al0., 1996). The cell death modes of cardiomyocytes during I/R injury are various, and the cause of cell death owning to CaMKII may be dependent on the accumulation of intracellular Ca^2+^. In apoptosis, Ca^2+^ accumulation is relatively slow in apoptosis and other forms of programmed cell death (mainly necroptosis) (Wang et al., 2010). In 2006, by modulating myocardial CaMKII through the expression of highly specific CaMKII inhibitory peptide AC3-I, researchers showed that AC3-I mice had reduced ER Ca ^2+^ content and were resistant to apoptosis induced by isoproterenol (ISO) and MI, suggesting that inhibition of CaMKII or ER Ca^2+^ leakage prevents cardiomyocyte apoptosis in pathological condition (Yang et al., 2006). CaMKII inhibition reduced caspase-3 activation and the number of TUNEL-positive cells and increased the Bcl-2/Bax ratio (Vila-Petroff et al., 2007). As the upstream of CaMKII, β1-AR, as a common target in I/R injury or LPS stimulation, induces both apoptosis and necrosis of cardiomyocytes (Yoo et al., 2009; Wang et al., 2015). PLN and RyR2, two major activated CaMKII downstream substrates on ER, mediate apoptosis in myocytes. In PLN double-mutants mice and constitutive RyR2 activation mice (RyR2 S2814D mice), the infarct size increased and myocardial apoptosis happened after I/R damage (Di Carlo et al., 2014). Moreover, apoptosis was reduced in CaMKIIδ-knockout mouse in the TAC model, maybe due to Akt inactivation (Toischer et al., 2010). For drugs inhibition, estrogen inhibits CaMKII expression by protein kinase A (PKA), thereby alleviating ISO-induced cardiac I/R injury (Ma et al., 2009).

### Calmodulin-Dependent Protein Kinase II and Necrosis

The previous study has demonstrated that the necrosis of myocytes, which is often accompanied by other cell death mode (for example apoptosis), is one of the reasons for the progressive loss of myocardial cells after MI despite its small proportion in cardiomyocyte’s death after MI (Kajstura et al., 1996). When the accumulation rate of Ca^2+^ is relatively fast in cardiomyocyte (Ca^2+^ overload occurs rapidly), ER uptakes and releases more Ca^2+^, resulting to the hyper-contraction of myofilament and mitochondrial hyperpermeability (Garcia-Dorado et al., 2012), thus leads to mPTP and MCU opening and loss of mitochondrial membrane potential (mitoptosis) (Joiner et al., 2012). With the dysfunction of mitochondria, ATP cannot be produced and necrosis happens (Zhu et al., 2021). Thus few studies have been conducted on myocardial necrosis due to the relatively moderate intracellular Ca^2+^ accumulation during I/R injury. From the perspective of molecular biology, CaMKII inhibition reduces LDH release, suggesting that CaMKII inhibition could prevent necrosis process (Vila-Petroff et al., 2007). As the upstream of CaMKII, the activation of β1-AR induces both apoptosis and necrosis of cardiomyocytes during I/R (Yoo et al., 2009). Furthermore, phosphorylation of PLN and RyR2, two major activated CaMKII downstream substrates on ER, mediates necrosis of cardiomyocytes. Constitutive activation of RyR2 phosphorylation site S2814 gives rise to the increase of infarct size after I/R, myocardial necrosis happened, while the lack of PLN activation can also aggravate myocardial I/R injury, suggesting that CaMKII mediates necrosis of myocardial cells through phosphorylation on RyR2 (Di Carlo et al., 2014). For PLN, its activation involves the reverse of ER Na^+^–Ca^2+^ exchanger (NCX) pattern, then induces a cascade to mitochondrial effector like MCU or mPTP to induce mitoptosis,^27 35^ a cell death mode was often classified into necrosis. Until now, more pathways and chemical substrates are related to CaMKII and mitoptosis in myocardial I/R (Wang J. et al., 2021). As the development of biotechnology, a new mode of programmed cell death, necroptosis, has been reported (Zhou et al., 2016).

### Calmodulin-Dependent Protein Kinase II and Pyroptosis/Inflammatory-Related Cell Death

Pyroptosis has been widely reported in cell and organ injury, and its mechanism has been gradually clarified in recent 5 years. In brief, pyroptosis is an under-controlled programmed inflammatory death mediated by caspase-1, -4, -5, and its effectors gasdermin family (Shi et al., 2017). The classical pathway of pyroptosis is the activation of inflammasome; the most common is NLRP3 inflammasome (He et al., 2016). There are more research studies focused on MI and pyroptosis; however, there is still no relevant report linking CaMKII and cardiomyocyte pyroptosis (cell perforation by gasdermin family) except several research studies focused on the activation of NLRP3 inflammasome by CaMKII. Inflammasomes were activated prior to Ang II-induced cardiomyocyte death in the normal mice group and weakened in CaMKIIδ knockout mice, and inflammasomes can recruit immune cells like macrophage to infiltrate myocardium, causing the cardiac remodeling (Willeford et al., 2018). Pression overload acts as the induction of NLRP3 inflammasome, which is accompanied by the expression of inflammatory genes (Suetomi et al., 2018; Suetomi et al., 2019). Moreover, the blockage of calpain (CAPN) could inhibit the NLRP3/ASC/caspase-1 pyroptosis pathway in the hypoxia-reoxygenation process of cardiomyocytes (Yue et al., 2019), while CAPN is closely related to CaMKII. In I/R, calpain binds to phosphorylated CaMKII and promotes the transport of phosphorated CaMKII and CapN1 to the ER membrane, thus activating the downstream receptor RyR2 (Lu et al., 2020). Despite this programmed inflammatory cell death mode, pyroptosis, CaMKII also mediates I/R injury through activation of the NF-κB inflammatory pathway. CaMKIIδ depletion attenuates I/R-induced inflammation and upregulated nuclear factor-κB (NF-κB); meanwhile, its activation is independent of cardiomyocyte necrosis. The expression of activated CaMKII in cardiomyocytes contributes to phosphorylation of IκB kinase (IκK) and the increase of nuclear factor p65, suggesting that CaMKII may activate NF-κB through IκK during I/R (Ling et al., 2013). Further studies showed that selective activation of CaMKIIδC during I/R was more likely to activate NF-κB and expressed more TNF-α compared with activation of CaMKIIδB, suggesting the acute activation of CaMKIIδC and NF-κB in reperfusion pathogenesis (Gray et al., 2017). In addition, myocardial knockdown of CaMKIIδ significantly reduces the activation of NF-κB, the expression of inflammatory chemokines and cytokines in Ang II infusion, while with the infusion of Ang II, CaMKII-dependent inflammatory gene expression and inflammasome development could be detected before the recruitment of macrophage, ultimately brings about cardiac fibrosis (Willeford et al., 2018). CaMKIIδC can also induce cardiomyocyte to express pro-inflammatory chemokine signal like macrophage inflammatory protein 1 (MIP-1) in post-MI through the inflammatory pathway rather than induction of cell death in post-MI early state and mediate changes in immune cells infiltration and cardiac remodeling, suggesting that CaMKII modulates post-pathological infarction (Weinreuter et al., 2014). The expression of inflammatory genes can also be triggered by activated CaMKII in cardiomyocytes in the mice TAC model, while by knocking out CaMKIIδ, inhibiting monocyte chemotactic protein-1 (MCP-1), and suppressing inflammasome are able to effectively reverse cardiac remodeling (Suetomi et al., 2018; Suetomi et al., 2019). Moreover, a critical protein in the inflammatory pathway, MyD88, also triggers cardiac hypertrophy and cardiomyocyte death in MI through oxidation of CaMKII (Singh et al., 2012). To inactivate NLRP3 inflammasome and inhibit the inflammatory response, some common drugs are useful. PCSK9 inhibitor and statin can both inactivate NLRP3 inflammasome (Wang X. et al., 2020; Chen et al., 2021), while melatonin can downregulate CaMKII in isolated heart I/R injury under intermittent hypoxia condition; by this way, it reduces the release of inflammatory factors like TNF-α and IL-6 (Yeung et al., 2015). During I/R, SGLT2 inhibitors effectively reduce the inflammatory response and the formation of inflammasomes in cardiomyocytes, and the mechanism may be related to the downregulation of CaMKII and the activation of phosphorylated AMPKα by maintaining intracellular ion homeostasis (Andreadou et al., 2020). Thus, CaMKII simultaneously activates NLRP3 inflammasomes, induces inflammatory response through the NF-κB inflammatory pathway, and mediates the involvement of immune cells in post-MI remodeling.

### Calmodulin-Dependent Protein Kinase II and Necroptosis

Necroptosis is a newly found cell death mode, which the death cell’s morphology resembles between necrosis and apoptosis, and it acts as a critical role in cell survival and diseases (Weinlich et al., 2017). The main pathway in necroptosis includes the inhibition of caspase-8, resulting in activation of receptor-interacting protein (RIP) kinase family activation (RIP1 and RIP3); as two substrates of effector protein mixed lineage kinase domain-like protein (MLKL), it is phosphorylated and oligomerized to perforate the cell membrane (Weinlich et al., 2017). It was found that RIP3, rather than RIP1, activates CaMKII to trigger cardiomyocyte necroptosis by mPTP (Zhang et al., 2016). This study verified that RIP3-induced phosphorylation or oxidative activation of CaMKII triggers the opening of the mPTP and myocardial necrosis in HF due to I/R or doxorubicin (Zhang et al., 2016). Further studies showed that in the ischemic preconditioning of the rat heart, the RIP1 inhibitor NEC-1 and its combination improved the recovery of ischemic cardiac function and reduced the infarction area by preventing MLKL oligomerization and translocation to the membrane. It is suggested that inhibition of necroptosis plays an important role in cardioprotecion in ischemic preconditioning independent of CaMKII signal transduction and oxidative stress (Szobi et al., 2018). Some drugs or inhibitors have effect on the necroptosis pathway in MI or I/R. Melatonin attenuates chronic pain–related MI susceptibility by inhibiting the RIP3-MLKL/CaMKII signaling pathway (Yang et al., 2018), and it can also alleviate endothelial necroptosis by the RIPK3-PGAM5-CypD-mPTP axis in cardiac microvascular I/R injury (Zhou et al., 2018). ZYZ-803, as a compound producing NO and hydrogen sulfide gas, can both alleviate ER stress and necroptosis after MI by suppression of the RIP3-CaMKII pathway (Chang et al., 2019). Adenosine kinase (ADK) inhibitor in I/R injury results in diminishing of CaMKII and MLKL phosphorylation; in addition to stabilizing the X-linked apoptotic protein (XIAP), it inhibits both necroptotic and apoptotic pathways during I/R (Yoo et al., 2009). Bisphenol A upregulates the RIPK3/CaMKII pathway in coronary endothelial cells to decline the integration of artery wall by the necroptotic pathway (Reventun et al., 2020). Necroptosis also occurs in other cardiac injuries such as hyperglycemic myocardial dysfunction (Sun et al., 2019), and it may also provide a new direction in I/R and ischemic cardiomyopathy in the future.

### Calmodulin-Dependent Protein Kinase II and Autophagy

Although autophagy and CaMKII have been studied deeply in neurology and oncology (Jing et al., 2016; Li et al., 2017), they are still less known in myocardial I/R injury. In the mouse HF model, the activation of caspase-3 could not be detected in a small portion of TUNEL-positive cardiomyocytes. However, autophagic death was only found in approximately 0.3% of cardiomyocytes in ischemic or dilated cardiomyopathy (Knaapen et al., 2001). Autophagy-induced death was confirmed by cytoplasmic inclusion body called autophagic body. Thus, this group of autophagic cardiomyocytes is characterized by granular cytoplasmic ubiquitin inclusions, but both necrosis and apoptosis markers, like TUNEL stain and caspase-9, are negative; meanwhile, caspase-3 and -7 cleavage are also absent (Knaapen et al., 2001). It was reported that ROS activates the TRPM2-Ca^2 +^-CaMKII cascade to phosphorylate Beclin1 on Ser295, thus leading to autophagy inhibition (Wang et al., 2016). However, it remains to be studied whether ROS affects autophagy through related pathways during cardiac I/R. And inhibiting CaMKII by KN-93 in the cardiac remodeling model induced by free fatty acid and hyperlipidemia, the autophagy level is decreased, which demonstrates a potential path to prevent fat-induced myocardial remodeling (Zhong et al., 2017). Recently, research demonstrated that inhibition of CaMKIIδ decreases beclin-1 phosphorylation at Ser90, which reduces myocardial autophagy and I/R damage, while beclin-1 siRNA has little effect on CaMKII phosphorylation (Kong et al., 2020). In addition, CaMKIIδC upregulates the expression of class I histone deacetylase (HDAC) in HF, including HDAC1 and HDAC3, but only HDAC1 inhibitors downregulate the autophagy gene of cardiomyocytes and reduce autophagic death of cardiomyocytes (Zhang et al., 2020). While applying traditional Chinese medicine *Panax Notoginseng* saponins (PNS), the cardiac function was reserved after MI by phosphorylating CaMKII and its downstream AMPK (Wang D. et al., 2021), which possibly through AMPK/mTOR signaling to activate not only cardiomyocyte but also vascular smooth muscle cell (VSMC) and endothelial cell autophagy (Hughes et al., 2020). Thus, both excessive activation and excessive inhibition of autophagy give rise to cardiomyocyte death, and CaMKII plays a certain role in these processes.

### The Role of Calmodulin-Dependent Protein Kinase II and Other Modes of Cardiomyocyte Death

**FIGURE 4:**
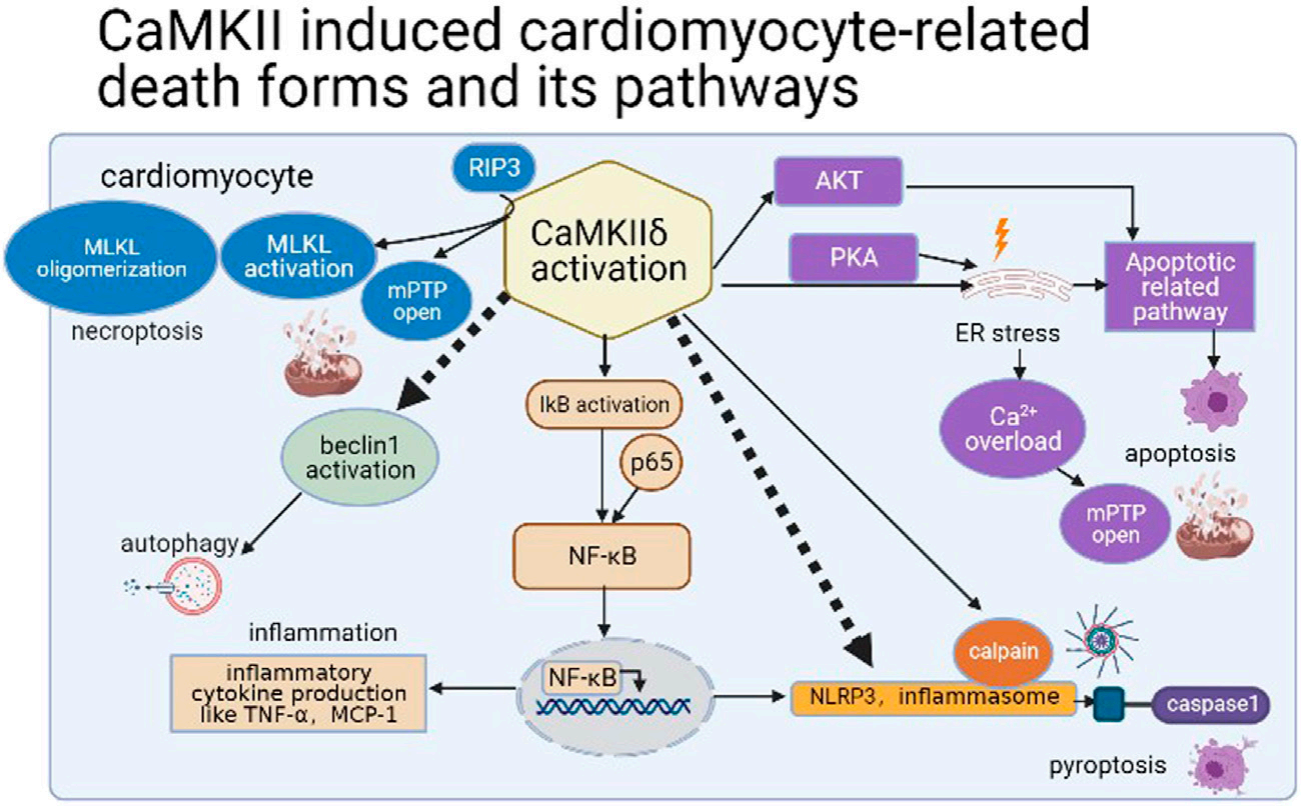
CaMKII induced cardiomyocyte-related death forms and its pathways.

Ferroptosis (Li et al., 2021), parthanatos, and other cardiomyocyte death forms (Del Re et al., 2019) are also included in a variety of cardiac pathological conditions. Among them, ferroptosis is associated with the accumulation of ion and lipid, which can be induced by erastin (Tang et al., 2019). In diabetic myocardial I/R injury, ER stress leads to ferroptosis of myocardial cells; in addition, inhibition of ferroptosis can reduce the cardiotoxicity in I/R and doxorubicin-induced HF (Fang et al., 2019; Li et al., 2020). However, ER stress is closely related to the activation of CaMKII; thus, we speculate that CaMKII activation may lead to ferroptosis of cardiomyocytes through ER stress. Furthermore, parthanatos is featured by hyper-activation of PARP-1 and accumulation of PARP in the cytosol and then leads to DNA fragmentation related by apoptosis-inducing factor (AIF) (Tang et al., 2019). In 2017, it was found that PARP was activated and AIF was translocated in circulating leukocyte in chronic HF patients (Bárány et al., 2017). But the relative mechanism and its existence in different cardiac diseases remain to be solved. Therefore, a variety of cell death modes occur during myocardial I/R and associate with CaMKII activation (Figure 4).

## Calmodulin-Dependent Protein Kinase II Inhibition

Since the first CaMKII inhibitor, KN-62 was invented in experiment (Tokumitsu et al., 1990); more and more drugs targeting CaMKII were discovered. Yet most of drugs are still utilized in animal models, only three specific CaMKII inhibitors, rimacalib (SMP-114), tatCN21 (Zybura et al., 2020), and ranolazine are applied in clinical trials, so there is still a long journey to the development of CaMKII-related medicine and its utilization in myocardial I/R injury. Meanwhile, many commonly used drugs have effect on CaMKII suppression, which is also unfavorable to the passion of targeting CaMKII invention. In this article, we enumerate several CaMKII-specific inhibitors (Table 2) and drugs affecting CaMKII and its related pathway in I/R (Table 3).

**TABLE 2:** Related drugs targeting CaMKII in disease.

CaMKII inhibitor	Subject	Clinical trial	Disease treatment	Reference
KN-93	Rabbit and rat	No	Hypokalemia-induced ventricular arrhythmia	Pezhouman et al. (2015)
KN-62	Rat	No	Heart I/R injury	Lu et al. (2020)
AIP	Rat	No	Diabetes heart disease	Daniels et al. (2018)
Rimacalib (SMP-114)	Human	Yes	Rheumatoid arthritis (RA); phase 2, NCT00296257; and ventricular arrhythmia	Neef et al. (2017); Westra et al. (2009)
RA608	Human and mouse	No	Arrhythmia and HF	Mustroph et al. (2020)
Ranolazine	Human and canis	Yes	Ventricular arrhythmia and related death, chronic kidney disease–induced arrhythmia, MI, and phase 2, NCT02360397	Zareba et al. (2018); Ke et al. (2020); Le et al. (2020)
PaAIP2	Mouse and rat	No	Neuronal dysplasticity	Murakoshi et al. (2017)
CN21(LY900014)/tatCN21	Human	Yes	Type 2 diabetes mellitus and Phase 3, NCT04605991	Tao-Cheng et al. (2013)
RA306	Rat and mouse	No	HF	Beauverger et al. (2020)

**TABLE 3:** Drugs or inhibitors affecting CaMKII-related pathways in I/R.

Drug/inhibitor	Subject and I/R method	Function	Related molecular and pathway	The inhibition of death pathway	Result after medication	Reference
Melatonin	1. SD rat, *ex vivo* I/R, and chronic intermittent hypoxia	1. Maintain ER Ca^2+^ homeostasis and enhance antioxidant enzyme activity	1. Unknown	1. Inflammatory response	1. Inflammation and fibrosis improved	Yeung et al. (2015); Zhou et al. (2018)
	2. Mouse *in vivo* I/R	2. Attenuation I/R-triggered microvascular necroptosis	2. RIPK3-PGAM5-CypD-mPTP pathway	2. Necroptosis	2. Reduce endothelial necroptosis	
SGLT2 inhibitor	Rat and mouse with many studies	Maintaining intracellular ion homeostasis, inhibiting reactive oxygen species, and AMPKα activation	Unknown, maybe AMPK activation	Inflammatory response, stress, and oxidation	I/R MI area maintained in the short term but decreased in the long term	Andreadou et al. (2020)
3, 4-dihydroxy flavonol	Rat *in vivo* I/R	Enhance the respiratory function and decrease the ROS production	Inhibit mPTP open	Mitoptosis	Preservation of mitochondrial function	Woodman et al. (2014)
Melatonin	Mouse *in vivo* I/R	Inhibition of RIP3 maybe	RIP3-MLKL/CaMKII pathway	Necroptosis and inflammatory response	Myocardial necrosis and ROS production were improved	Yang et al. (2018)
ZYZ-803	Mouse *in vivo* I/R	Hydrogen sulfide and nitric oxide are produced to maintain intracellular endoplasmic reticulum stability and influence necroptosis pathways	RIP3/CaMKII pathway	Necroptosis and ER stress	Reduce infarct size and improve cardiac function	Chang et al. (2019)
Total saponins of *Panax notoginseng*	Mouse *in vivo* I/R	Enhancing glucose deprivation induces autophagy, antiplatelet aggregation, angiogenesis, and endothelial migration	AMPK and CaMKII phosphorylation	Induction of autophagy	Enhance endothelial cell migration and angiogenesis	Wang D. et al., (2021)

## Conclusion

Cardiomyocyte Ca^2+^ overload is a major cause of I/R injury, initiating a cascade of events culminating in cardiomyocyte death and myocardial dysfunction. CaMKII activation is a key feature of myocardial I/R, leading to adverse reactions such as intracellular mitochondrial swelling, ER Ca^2+^ leak, and abnormal contraction of myofilaments. CaMKIIδ has been widely studied in the activation, localization, signaling pathways, and induced cell death of cardiomyocytes. Although the utilization of CaMKII inhibitor has not been carried out on a large scale in clinical work, many teams have made a great deal of contributions to relevant studies, and based on this theoretical basis, new targets and feasible inhibitors of related pathways have been sought. Understanding of CaMKII mode of action in cardiomyocytes death induced by I/R is helpful to ameliorate treatment strategies and find out new targets of CaMKII applied to new therapy. We anticipate that there will be several promising treatment regiments or drugs to correct abnormal activation of CAMKII in the future for I/R injury after MI and other related cardiac diseases.

## Glossary

ADK: Adenosine kinase
AngII: Angiotensin II
AMPK: Adenosine monophosphate-activated protein kinase
CaM: Calmodulin
CaMKII: Ca^2+^/CaM-dependent protein kinase ll
CaMKIIα: Ca^2+^/CaM-dependent protein kinase ll subtype α
CaMKIIβ: Ca^2+^/CaM-dependent protein kinase ll subtype β
CaMKIIγ: Ca^2+^/CaM-dependent protein kinase ll subtype γ
CaMKIIδ: Ca^2+^/CaM-dependent protein kinase ll subtype δ
CaMKIIδ9: Ca^2+^/CaM-dependent protein kinase ll subtype δ variant 9
CaMKIIδA: Ca^2+^/CaM-dependent protein kinase ll subtype δ variant A
CaMKIIδB: Ca^2+^/CaM-dependent protein kinase ll subtype δ variant B: Ca^2+^/CaM-dependent protein kinase ll subtype δ variant C
CaMKIIδB: Ca^2+^/CaM-dependent protein kinase ll subtype δ variant B: Ca^2+^/CaM-dependent protein kinase ll subtype δ variant C
CAPN: Calpain
ER: Endoplasmic reticulum
HDAC: Histone deacetylase
HF: Heart failure
I/R: Ischemia-reperfusion injury
ISO: Isoproterenol
JNK2: c-Jun N-terminal kinase 2
KATP: ATP-sensitive potassium channel
LTCC: L-type calcium channel
MCP1: Monocyte chemotactic protein 1
MCU: Mitochondrial calcium unidirectional transporter
MI: Myocardial infarction
MLKL: Mixed-lineage kinase domain-like protein
mPTP: Mitochondrial permeability transition pore
MsrA: Methionine sulfone reductase A
NCX: Sodium–calcium exchanger
NF-κB: Nuclear factor–κB
NLRP3: Nod-like receptor protein 3
NLS: Nuclear localization sequence
NO: Nitric oxide
OGA: O-GlcNAcase
O-GlcNAc: O-linked N-acetylglucosamine
OGN: O-GlcNAcylation protein
OGT: O-GlcNAc transferase
PHD2: Proline hydroxylase 2
PLN: Phospholamban
PKA: Protein kinase A
RIP1: Receptor-interacting protein 1
RIP3: Receptor-interacting protein 3
ROS: Reactive oxygen species
RyR2: Ryanodine receptor 2
SerCa2: Sarcoplasmic reticulum Ca∼(2+)-ATPase2
TNF-α: Tumor necrosis factor-α
ULK1: UNC-51–like kinase 1
XIAP: X-linked apoptotic protein inhibitor
β1-AR: β1-adrenergic receptor
